# An Adaptive Moment estimation method for online AUC maximization

**DOI:** 10.1371/journal.pone.0215426

**Published:** 2019-04-23

**Authors:** Xin Liu, Zhisong Pan, Haimin Yang, Xingyu Zhou, Wei Bai, Xianghua Niu

**Affiliations:** 1 College of Command and Control, Army Engineering University of PLA, Nanjing, China; 2 College of Communication Engineering, Army Engineering University of PLA, Nanjing, China; 3 Beijing Institute of Applied Meteorology, Beijing, China; HEC Montréal, CANADA

## Abstract

Area Under the ROC Curve (AUC) is a widely used metric for measuring classification performance. It has important theoretical and academic values to develop AUC maximization algorithms. Traditional methods often apply batch learning algorithm to maximize AUC which is inefficient and unscalable for large-scale applications. Recently some online learning algorithms have been introduced to maximize AUC by going through the data only once. However, these methods sometimes fail to converge to an optimal solution due to the fixed or rapid decay of learning rates. To tackle this problem, we propose an algorithm AdmOAM, Adaptive Moment estimation method for Online AUC Maximization. It applies the estimation of moments of gradients to accelerate the convergence and mitigates the rapid decay of the learning rates. We establish the regret bound of the proposed algorithm and implement extensive experiments to demonstrate its effectiveness and efficiency.

## Introduction

AUC [1] plays an important role in measuring classification performance, and quantifies the ability of a classifier that assigns a higher score for a randomly chosen positive instance than a randomly drawn negative instance [2]. Compared with accuracy and cross-entropy loss, AUC is independent of the priori class probability distribution and misclassification costs, which makes it more favourable for imbalanced classification tasks [3–6]. Moreover, AUC is largely applied in many real-world scenarios like cancer diagnosis and anomaly detection [7, 8].

In recent decades, many batch learning algorithms [9–12] have been introduced to optimize AUC directly. Despite the success of these batch AUC optimization algorithms, they all require the whole training instances available before training. Besides, they update the model every epoch with all training instances. Therefore, it is not efficient and scalable for large-scale applications in batch learning setting. To address this challenge, online learning technique has been introduced to maximize AUC, which has been shown to be capable for large-scale scenarios [13–15]. The online learning methods update the model with only one instance each epoch. As a result, it is desirable to apply online leaning algorithms for handling large-scale streaming data which arrives sequentially.

However, the task of AUC optimization requires minimizing the sum of the losses between instances from different classes. Therefore, it is difficult to maximize AUC by directly applying online learning, which requires to obtain all previous training instances at current iteration for calculating the sum of pairwise losses. Several recent works [16–19] adopt different approximations of the sum of pairwise losses to avoid storing all received training data, which makes them more feasible for large-scale tasks. In general, there are two kinds of online AUC maximization frameworks. The first framework uses reservoir sampling method that keeps fixed buffers to store some historical instances for calculating pairwise losses [18, 19]. The other framework employs one-pass technique to maximize AUC by processing each instance only once [17]. The work [16] proposed an *adaptive one-pass online AUC maximization* algorithm called AdaOAM. This method adjusts the learning rates of different dimensions to the geomerty of data by applying an *adaptive gradient method* (Adagrad) [20].

Despite AdaOAM has achieved good performance, its learning rate may shrink too fast due to the rapid increase of its denominators. As a result, the model may fail to fully converge [21]. To tackle this problem, we propose an algorithm AdmOAM, Adaptive Moment estimation method for Online AUC Maximization. It applies the estimation of moments of gradients to adaptively calculate the learning rates dimensionally based on the framework of [22]. Our method mitigates the rapid decay of learning rates by using exponential moving averages of past gradients as the denominators. Furthermore, AdmOAM is efficient and only requires to store the first and second moments of gradients. Based on the theoretical analysis of AdmOAM, we have found that the regret bound of AdmOAM stays much lower than existing non-adaptive methods and is comparable with AdaOAM. We have also shown the effectiveness of the proposed AdmOAM in experiments on several benchmark datasets in comparison with 4 state-of-the-art online AUC maximization algorithms.

The rest of the paper is organized as follows. We first give an overview of some related works. Then we describe the problem setting and the framework of AdmOAM. We then give the theoretical analysis and provide the experimental results. Finally, we present a summary and some directions for future work.

## Related work

In this section, we briefly review three prior works in related topics: online learning, adaptive gradient methods and AUC maximization.

### Online learning

Online learning is a type of efficient and scalable machine learning algorithm that updates the model from data sequentially. Compared to the traditional batch or offline learning algorithms, online learning algorithms avoid the time-consuming training process and are capable of retraining efficiently at the arrival of new data [13]. The first online learning algorithm is the Perceptron [23], which updates the model with the first-order gradient information. Passive-Aggressive is another type of first-order online algorithm, which applys the margin-based technique [24]. In recent years, several second-order online learning algorithms have been proposed to accelerate the convergence of optimization [25, 26]. Besides, some regularization terms have been introduced to stabilize the online learning models [11, 27]. However, these traditional online approaches are aimed at optimizing the classification accuracy or error rate, which are inappropriate for imbalanced classification tasks. In contrast, we develop a novel first-order online algorithm by maximizing a imbalanced metric with adaptive gradient method.

### Adaptive gradient methods

Online Gradient Descent (OGD) [28] is the dominant method for solving the online convex optimization problems. It updates a model by moving the parameters along the direction opposite the gradient of the loss function with a global learning rate. However, infrequently occurring features are highly informative and require relatively larger learning rates than frequently occurring features. Therefore, OGD can not fully incorporate the knowledge of the geometry of the data with the global learning rate. To tackle this challenge, some researchers have proposed several variants of OGD that perform adaptive gradient optimization by adjusting the learning rates on a per-feature basis iteratively [20, 22, 29]. The most famous adaptive gradient algorithm is Adagrad [20], which can achieve better performance than non-adaptive algorithms both theoretically and experimentally. However, Adagrad has been observed to diverge due to the rapid decay of the learning rates since the denominators of the learning rates are based on the accumulation of the square of the past gradients. To address this problem, some variants of Adagrad have been proposed, such as RMSprop [29], Adam [22] and AMSgrad [30]. These methods use exponential moving average to estimate the moments of the gradients, which can mitigate the rapid decay of the learning rates. Among these variants of Adagrad, Adam is the most widely used method due to its fast convergence and easiness in implement. Furthermore, Adam has been successfully used in many real-world applications like computational biology [31], automated driving [32], text categorization [33], machine translation [34], etc.

### AUC maximization

AUC has been widely used to evaluate the classification performance. Therefore, several algorithms have been proposed to maximize AUC with different convex surrogate losses [10, 16, 17, 19, 35]. Initially, many efforts have been devoted to optimize AUC in batch learning setting [10, 35]. However, those batch algorithms fail to meet the demands of efficient and scalable learning for large-scale tasks. Therefore, some online AUC maximization algorithms have been proposed [16, 17, 19]. Generally, there are two main online learning frameworks for AUC maximization. The first framework stores several fixed-size buffers and adopts the reservoir sampling technique to update the buffers for representing the received instances. The sizes of these buffers are related to the number of training instances that makes this type of algorithms impractical for large-scale applications. Besides, this framework use hinge losses as the surrogate loss, which has been proven to be inconsistent with AUC [36]. To overcome these limitations, the work [17] proposed a new framework called OPAUC, which applies the square loss as the surrogate loss function in one-pass learning setting. OPAUC utilizes the consistency between the square loss and the AUC score, and only maintains the mean vector and variance matrix of the received instances. Compared to the first framework, the storage requirement of OPAUC is independent of the number of training instances and each instance only requires to go through only once. But the above two frameworks are both based on the OGD for optimzation, which prevents them from exploiting the geometrical information of data [20]. For performing more informative gradient-based learning, the work [16] proposed an algorithm AdaOAM by applying one-pass framework and Adagrad [20].

Although AdaOAM has achieved fairly good performance, it may not fully converge according to the fact that the denominators of its learning rates are the accumulation of all previous gradients. The learning rates of AdaOAM would shrink fast with the rapid increase of the denominators, and this degrades its learning performance. To solve this problem, we develop a novel adaptive online AUC maximization algorithm called AdmOAM, which uses the square loss function in one-pass framework and applies Adam [22] for mitigating the rapid decay of the learning rates.

## Method

In this section, we present the framework of AdmOAM. We first introduce the problem setting of the online AUC maximization tasks. Then we present the details of AdmOAM.

### Problem setting

We concentrate on learning a linear model $f:R^{d}\to R$ in binary classification setting. We denote $X=R^{d}$ and Y={+1,-1} as the feature space and label space, respectively. Let D denotes an unknown distribution over *X* × *Y*. Let S denotes a sample that is drawn i.i.d from *D*. Let H denotes the hypothesis class. At the t-th iteration, we denote the received training instance as $(x_{t},y_{t})\in S$, and $w_{t}\in H$ is the linear model we learned currently. Let $S_{+}=\left\{\left(x_{i}^{+},+1\right)|i\in \left[n_{+}\right]\right\}$ be the set of positive instances and $S_{-}=\left\{\left(x_{i}^{-},-1\right)|i\in \left[n_{-}\right]\right\}$ be the set of negative instances in sample S, where *n*_+_ and *n*_−_ refer to the numbers of positive and negative instances, respectively. Then the AUC score of the linear function *f* on sample S can be calculated as:

$$\begin{array}{c} \begin{array}{cc} AUC(f)= & \frac{\sum_{i=1}^{n_{+}}\sum_{j=1}^{n_{-}}\left(I\left[f\left(x_{i}^{+}\right)>f\left(x_{j}^{-}\right)\right]+\frac{1}{2}I\left[f\left(x_{i}^{+}\right)=f\left(x_{j}^{-}\right)\right]\right)}{n_{+}n_{-}}\\ = & \frac{\sum_{i=1}^{n_{+}}\sum_{j=1}^{n_{-}}\left(I\left[w^{⊤}x_{i}^{+}>w^{⊤}x_{j}^{-}\right]+\frac{1}{2}I\left[w^{⊤}x_{i}^{+}=w^{⊤}x_{j}^{-}\right]\right)}{n_{+}n_{-}} \end{array} \end{array}$$ (1)

where **w** is the weight vector of function *f* and I[·] is the indicator function which outputs 1 if condition is satisfied and 0 otherwise. Practically, we use the least square loss $\ell \left(w^{⊤}\left(x_{i}^{+}-x_{j}^{-}\right)\right)={\left(1-w^{⊤}\left(x_{i}^{+}-x_{j}^{-}\right)\right)}^{2}$ as a surrogate of the indicator function. The square loss function is convex and keeps consistent with AUC [17]. Then we can minimize the following objective function for finding the optimal linear classifier.

$$\begin{array}{c} \begin{array}{cc} L(w)= & \frac{\lambda }{2}{\left\|w\right\|}_{2}^{2}+\frac{\sum_{i=1}^{n_{+}}\sum_{j=1}^{n_{-}}\ell \left(w^{⊤}\left(x_{i}^{+}-x_{j}^{-}\right)\right)}{2n_{+}n_{-}}\\ = & \frac{\lambda }{2}{\left\|w\right\|}_{2}^{2}+\frac{\sum_{i=1}^{n_{+}}\sum_{j=1}^{n_{-}}{\left(1-w^{⊤}\left(x_{i}^{+}-x_{j}^{-}\right)\right)}^{2}}{2n_{+}n_{-}} \end{array} \end{array}$$ (2)

where $\frac{\lambda }{2}{\left\|w\right\|}_{2}^{2}$ is introduced as the regularizer to reduce the complexity of the linear classifier. Next we present the details of AdmOAM.

### Adaptive moment online AUC maximization

In online learning framework, we focus on minimizing the regret of a sequence of algorithms with regard to a competing hypothesis, where the model of the competing hypothesis is the optimal decision in hindsight. The optimal decision w* is defined as:

$$\begin{array}{c} w^{*}=\underset{w\in \chi }{\text{arg}\;\text{min}}\sum_{t=1}^{T}f_{t}\left(w\right), \end{array}$$ (3)

where *χ* is the decision set.

The regret of hypothesis H at iteration T∈N is defined as:

$$\begin{array}{c} Regret_{T}\left(H\right)=\sum_{t=1}^{T}f_{t}\left(w_{t}\right)-\sum_{t=1}^{T}f_{t}\left(w^{*}\right). \end{array}$$ (4)

where $w_{t},w^{*}\in H$. According to the approach in [17], the overall loss L(w) can be transformed to a sum of losses on each training instance in online setting

$$\begin{array}{ccc} L(w) & = & \sum_{t=1}^{T}L_{t}\left(w\right)\;where\\ L_{t}\left(w\right) & = & \frac{\lambda }{2}{\left\|w\right\|}_{2}^{2}+\frac{\sum_{i=1}^{t-1}I\left[y_{i}\neq y_{t}\right]{\left(1-y_{t}w^{⊤}\left(x_{t}-x_{i}\right)\right)}^{2}}{2|\{i\in \left[t-1\right]:y_{i}y_{t}=-1\}|} \end{array}$$ (5)

where $S_{t}=\left\{\left(x_{i},y_{i}\right)|i\in \left[t\right]\right\}$ denotes the i.i.d training sample on the *t*-th iteration, and $L_{t}\left(w\right)$ is an unbiased estimation of L(w). Then the gradient of $L_{t}\left(w\right)$ can be calculated as:

$$\begin{array}{c} \nabla L_{t}\left(w\right)=\{\begin{array}{cc} \lambda w+x_{t}x_{t}^{⊤}w-x_{t}+\frac{\sum_{i:y_{i}=-1}\left[x_{i}+\left(x_{i}x_{i}^{⊤}-x_{i}x_{t}^{⊤}-x_{t}x_{i}^{⊤}\right)w\right]}{T_{t}^{-}} & \text{if}\;\;y_{t}=+1\\ \lambda w+x_{t}x_{t}^{⊤}w+x_{t}+\frac{\sum_{i:y_{i}=+1}\left[-x_{i}+\left(x_{i}x_{i}^{⊤}-x_{i}x_{t}^{⊤}-x_{t}x_{i}^{⊤}\right)w\right]}{T_{t}^{+}} & \text{if}\;\;y_{t}=-1 \end{array} \end{array}$$ (6)

where $T_{t}^{+}$ and $T_{t}^{-}$ denote the numbers of positive and negative instances in $S_{t}$, respectively. For calculating $\nabla L_{t}\left(w\right)$ without storing all received instances, we use **c**^±^ and Γ^±^ as the mean vectors and covariance matrices of positive and negative instances, respectively.

$$\begin{array}{c} c_{t}^{\pm }=\sum_{i:i<t,y_{i}=\pm 1}\frac{x_{i}}{T_{t}^{\pm }}\;and\;\Gamma_{t}^{\pm }=\sum_{i:i<t,y_{i}=\pm 1}\frac{x_{i}x_{i}^{⊤}-c_{t}^{\pm }{\left[c_{t}^{\pm }\right]}^{⊤}}{T_{t}^{\pm }} \end{array}$$ (7)

The mean vectors and covariance matrices can be updated as follows:

$$\begin{array}{c} \begin{array}{cc} c_{t}^{\pm }=\;c_{t-1}^{\pm } & +\frac{1}{T_{t}^{\pm }}\left(x_{t}^{\pm }-c_{t-1}^{\pm }\right)\\ \Gamma_{t}^{\pm }=\;\Gamma_{t-1}^{\pm }+c_{t-1}^{\pm }{\left[c_{t-1}^{\pm }\right]}^{⊤} & -c_{t}^{\pm }{\left[c_{t}^{\pm }\right]}^{⊤}+\frac{x_{t}^{\pm }{\left(x_{t}^{\pm }\right)}^{⊤}-\Gamma_{t-1}^{\pm }-c_{t-1}^{\pm }{\left[c_{t-1}^{\pm }\right]}^{⊤}}{T_{t}^{\pm }}. \end{array} \end{array}$$ (8)

Therefore, the gradient $\nabla L_{t}\left(w\right)$ can be reformulated as:

$$\begin{array}{c} \nabla L_{t}\left(w\right)=\{\begin{array}{cc} \lambda w-x_{t}+c_{t}^{-}+\left(x_{t}-c_{t}^{-}\right){\left(x_{t}-c_{t}^{-}\right)}^{⊤}+\Gamma_{t}^{-}w\; & \text{if}\;\;y_{t}=+1\\ \lambda w+x_{t}-c_{t}^{+}+\left(x_{t}-c_{t}^{+}\right){\left(x_{t}-c_{t}^{+}\right)}^{⊤}+\Gamma_{t}^{+}w\; & \text{if}\;\;y_{t}=-1 \end{array} \end{array}$$ (9)

Then we can update the linear classifier by using online gradient descent **w**_*t*+1_ = **w**_*t*_ − *η*_*t*_ **g**_*t*_, where *η*_*t*_ is the learning rate at iteration *t* and $g_{t}=\nabla L_{t}\left(w\right)$. According to the properities of strong convexity in [16], the optimal w_*_ is satisfied with $\|w_{*}{\|}_{2}\leq 1/\sqrt{\lambda }$. As a result, it is reasonable to restrict **w**_*t*_ with $\|{\text{w}}_{t+1}{\|}_{2}\leq 1/\sqrt{\lambda }$ by applying the projected gradient method [28].

$$\begin{array}{c} w_{t+1}=\Pi_{\frac{1}{\sqrt{\lambda }}}\left(w_{t}-\eta_{t}g_{t}\right)=\underset{{\left\|w\right\|}_{2}\leq 1/\sqrt{\lambda }}{argmin}{\left\|w-\left(w_{t}-\eta_{t}g_{t}\right)\right\|}_{2}^{2} \end{array}$$ (10)

However, it has been shown that the model can not fully exploit the geometrical information of data with a global learning rate [20]. For solving this problem, [16] proposed AdaOAM by updating the learning rates of different features as follows:

$$\begin{array}{c} \eta_{i}^{\left(t\right)}=\frac{\rho }{\sqrt{\epsilon +\sum_{k=1}^{t}g_{k,i}^{2}}} \end{array}$$ (11)

where *i* ∈ [*d*] is the *i*-th dimensional feature and *t* is the number of iterations.

**Algorithm 1** The AdmOAM Algorithm

**Input**: The regularization parameter λ > 0, the step size ${\left\{\eta_{t}\right\}}_{t=1}^{T}$, 1*st* moment (the mean) and 2*nd* raw moment (the uncentered variance), the exponential decay rates *β*_1_, *β*_2_ ∈ [0, 1) and a smooth parameter *ϵ* > 0.

**Output**: Updated classifier **w**_*T*_.

**Variables**: $m_{0},v_{0}\in R^{d}$.

**Initialize** $T_{0}^{\pm }=0,w_{0}=0,m_{0}=v_{0}=0,c_{0}^{\pm }=0,\Gamma_{0}^{\pm }={\left[0\right]}_{d\times d}$.

**for** *t* = 1, 2, …, *T* **do**

 Observe instance (**x**_*t*_, *y*_*t*_);

 **if** *y*_*t*_ = +1 **then**

  $T_{t}^{+}=T_{t-1}^{+}+1$, $T_{t}^{-}=T_{t-1}^{-}$;

  $c_{t}^{+}=c_{t-1}^{+}+\frac{1}{T_{t}^{+}}\left(x_{t}-c_{t-1}^{+}\right)$ and $c_{t}^{-}=c_{t-1}^{-}$;

  Update $\Gamma_{t}^{+}$ and $\Gamma_{t}^{-}=\Gamma_{t-1}^{-}$;

 **else**

  $T_{t}^{-}=T_{t-1}^{-}+1$, $T_{t}^{+}=T_{t-1}^{+}$;

  $c_{t}^{-}=c_{t-1}^{-}+\frac{1}{T_{t}^{-}}\left(x_{t}-c_{t-1}^{-}\right)$ and $c_{t}^{+}=c_{t-1}^{+}$;

  Update $\Gamma_{t}^{-}$ and $\Gamma_{t}^{+}=\Gamma_{t-1}^{+}$;

 **end if**

 Calculate gradient $g_{t}=\nabla L_{t}\left(w_{t-1}\right)$;

 Update **m**_*t*_, **v**_*t*_;

 ${\bar{m}}_{t}=m_{t}/\left(1-\beta_{1}^{T}\right),{\bar{v}}_{t}=v_{t}/\left(1-\beta_{2}^{T}\right)$;

 $w_{t}=\;\Pi_{\frac{1}{\lambda }}(w_{t-1}-\eta_{t}\cdot {\bar{m}}_{t}/\left(\sqrt{{\bar{v}}_{t}}+\epsilon \right))$;

**end for**

When the value of the accumulation of previous gradients increases too fast, the learning rate of AdaOAM would shrink to a much small value, which can result in a slow convergence. Therefore, we propose AdmOAM for alleviating the rapid decay of learning rates, and our work is inspired by [22]. For the learning rates of different features, AdmOAM adaptively updates them with the estimations of moments of gradients. By using the exponential moving averages of previous gradients as the denominators, AdmOAM mitigates the rapid decay of learning rates.

Specifically, we denote **m** and **v** as the exponential moving averages of the gradients and the squared gradients, respectively. These two vectors are introduced as the estimations of the first and second moments of gradients. They can be updated as follows:

$$\begin{array}{c} \begin{array}{cc} m_{t}= & \;\beta_{1}\cdot m_{t-1}+\left(1-\beta_{1}\right)\cdot g_{t}\\ v_{t}= & \;\beta_{2}\cdot v_{t-1}+\left(1-\beta_{2}\right)\cdot g_{t}^{2} \end{array} \end{array}$$ (12)

where *β*_1_, *β*_2_ ∈ [0, 1) are the exponential decay rates of **m** and **v**. Due to the property of exponential moving average, the denominator would not shrink too fast. Besides, AdmOAM only requires extra *O*(*d*) space for storing **m** and **v** as compared to the efficient OPAUC. Note that if **m** and **v** are zero vectors in initialization, then the correction of the bias is needed according to [22]. Therefore, the classifier **w** with the initialization bias correction can be updated as:

$$\begin{array}{ccc} {\bar{m}}_{t} & = & \;m_{t}/\left(1-\beta_{1}^{T}\right)\\ {\bar{v}}_{t} & = & \;v_{t}/\left(1-\beta_{2}^{T}\right)\\ w_{t} & = & \;\Pi_{\frac{1}{\sqrt{\lambda }}}\left(w_{t-1}-\eta_{t}\cdot {\bar{m}}_{t}/\left(\sqrt{{\bar{v}}_{t}}+\epsilon \right)\right) \end{array}$$ (13)

where *ϵ* > 0 is a smooth parameter for preventing the denominator becoming zero. The framework of AdmOAM is shown in Algorithm 1.

## Theoretical analysis

Next we present our main theoretical results of AdmOAM.

**Lemma 1**. *Let* **w**_*t*_ *and* **g**_*t*_ (*t* ∈ [*T*]) *be the weight vector and gradient defined in the Algorithm 1, and we have*

$$\begin{array}{c} {\left(g_{t,i}\right)}^{2}\leq 2{\left(\lambda w_{t-1,i}\right)}^{2}+2{\left(1+\frac{\sqrt{\sum_{i=1}^{d}r_{t,i}^{2}}}{\sqrt{\lambda }}\right)}^{2}r_{t,i}^{2} \end{array}$$ (14)

where we denote the *i*-th dimension of the gradient at iteration *t* as **g**_*t*,*i*_ and *r*_*t*,*i*_ = *max*_*j*<*t*_|**x**_*j*,*i*_ − **x**_*t*,*i*_|.

*Proof*. Firstly, we define $w^{*}=\underset{w\in \chi }{argmin}\sum_{t}L_{t}\left(w\right)$ as the optimal weight vector of the linear model in hindsight. Objective function $L_{t}\left(w\right)$ uses $\frac{\lambda }{2}{\left\|w\right\|}_{2}^{2}$ as the regularizer. According to the strongly convex property, we have $\|w^{*}{\|}_{2}^{2}\leq 1/\lambda$ [16]. As a result, we restrict **w**_*t*_ with $\|w_{t}{\|}_{2}\leq 1/\sqrt{\lambda }$ by applying the projected gradient update rule. Besides, according to the definition of the gradient of $L_{t}\left(w_{t-1}\right)$, we have $g_{t}=\nabla L_{t}\left(w_{t-1}\right)$. If *y*_*t*_ = 1, we have

$$\begin{array}{ccc} {\left(g_{t,i}\right)}^{2} & = & [\lambda w_{t-1,i}+\frac{\sum_{j:y_{j}=-1}\left(1-y_{t}\left\langle x_{t}-x_{j},w_{t-1}\right\rangle \right)y_{t}\left(x_{j,i}-x_{t,i}\right)}{T_{t}^{-}}{]}^{2}\\ & = & [\lambda w_{t-1,i}+\frac{\sum_{j:y_{j}=-1}\left(1-\left\langle x_{t}-x_{j},w_{t-1}\right\rangle \right)\left(x_{j,i}-x_{t,i}\right)}{T_{t}^{-}}{]}^{2} \end{array}$$ (15)

where $T_{t}^{-}$ denotes the number of the received negative instances at iteration *t*. By applying inequality 〈**w**, **v**〉 ≤ ‖**w**‖_2_‖**v**‖_2_ and (*a* + *b*)^2^ ≤ 2*a*^2^ + 2*b*^2^, we have

$$\begin{array}{ccc} {\left(g_{t,i}\right)}^{2} & \leq & [\lambda |w_{t-1,i}|+\frac{\sum_{j:y_{j}=-1}\left(1+\right\|x_{t}-x_{j}{\|}_{2}\|w_{t-1}{\|}_{2})\|x_{j,i}-x_{t,i}{\|}_{2}}{T_{t}^{-}}{]}^{2}\\ & \leq & [\lambda |w_{t-1,i}|+\left(1+\frac{\sqrt{\sum_{i=1}^{d}r_{t,i}^{2}}}{\sqrt{\lambda }}\right)r_{t,i}{]}^{2}\\ & \leq & 2{\left(\lambda w_{t-1,i}\right)}^{2}+2{\left(1+\frac{\sqrt{\sum_{i=1}^{d}r_{t,i}^{2}}}{\sqrt{\lambda }}\right)}^{2}r_{t,i}^{2} \end{array}$$ (16)

This upper bound also holds for *y*_*t*_ = −1.

**Lemma 2**. *Assume the gradient of the objective function f*_*t*_ *is bounded, sup*_**w**∈*χ*_‖**g**_*t*_(**w**)‖_2_ ≤ *G*, *sup*_**w**∈*χ*_‖**g**_*t*_(**w**)‖_∞_ ≤ *G*_∞_ *and the distance between any elements of the hypothesis class is bounded, sup*_**w**,**u**∈*χ*_‖**w** − **u**‖_2_ ≤ *D*, *sup*_**w**,**u**∈*χ*_‖**w** − **u**‖_∞_ ≤ *D*_∞_ *and β*_1_, *β*_2_ ∈ [0, 1) *satisfy* $\gamma =\frac{\beta_{1}^{2}}{\sqrt{\beta_{2}}}\leq 1$. *Let* $\eta_{t}=\frac{\eta }{\sqrt{t}}$ *and β*_1,*t*_ = *β*_1_ *ρ*^*t*−1^, *ρ* ∈ (0, 1). *For any* *T* > 1, *the following regret bound holds*

$$\begin{array}{c} \begin{array}{cc} R(T)\leq & \frac{D^{2}}{2\eta (1-\beta_{1})}\sum_{i=1}^{d}\sqrt{T{\bar{v}}_{T,i}}+\frac{\eta \left(1+\beta_{1}\right)G_{\infty }}{\left(1-\beta_{1}\right)\sqrt{1-\beta_{2}}{\left(1-\gamma \right)}^{2}}\sum_{i=1}^{d}{\left\|g_{1:T,i}\right\|}_{2}\\ + & \sum_{i=1}^{d}\frac{D_{\infty }^{2}G_{\infty }\sqrt{1-\beta_{2}}}{2\eta \left(1-\beta_{1}\right){\left(1-\rho \right)}^{2}} \end{array} \end{array}$$ (17)

*where* **g**_1:*T*,*i*_ = [**g**_1,*i*_, **g**_2,*i*_, ⋯, **g**_*t*,*i*_].

Lemma2 is the Theorem 4.1 from the work [22]. Next we derive the regret bound of the proposed AdmOAM algorithm.

**Theorem 1**. *Assume* ‖**x**‖_∞_ ≤ 1, *β*_1_, *β*_2_ ∈ [0, 1) *satisfy* $\gamma =\frac{\beta_{1}^{2}}{\sqrt{\beta_{2}}}\leq 1$. *Let* $\eta_{t}=\frac{\eta }{\sqrt{t}}$ *and β*_1,*t*_ = *β*_1_ *ρ*^*t*−1^, *ρ* ∈ (0, 1). *For any T* > 1, *AdmOAM can achieve following regret bound*

$$\begin{array}{cc} R(T)\leq & A_{1}\frac{\sum_{i=1}^{d}\sqrt{T\sum_{j=1}^{T}\left(1-\beta_{2}\right)\beta_{2}^{T-j}B_{j,i}}}{\sqrt{1-\beta_{2}^{T}}}+A_{2}\sum_{i=1}^{d}\sqrt{\sum_{t=1}^{T}B_{j,i}}+A_{3} \end{array}$$ (18)

*where* $A_{1}=\frac{2}{\lambda \eta (1-\beta_{1})}$,$A_{2}=\frac{2\eta \left(1+\beta_{1}\right)\sqrt{\lambda +4{\left(1+2\sqrt{\frac{d}{\lambda }}\right)}^{2}}}{\left(1-\beta_{1}\right)\sqrt{1-\beta_{2}}{\left(1-\gamma \right)}^{2}}$, $A_{3}=\sum_{i=1}^{d}\frac{2\sqrt{2(1-\beta_{2})}\sqrt{\lambda +4{\left(1+2\sqrt{\frac{d}{\lambda }}\right)}^{2}}}{\eta \left(1-\beta_{1}\right){\left(1-\rho \right)}^{2}}$ *and* $B_{j,i}=2{\left(\lambda w_{j,i}\right)}^{2}+2{\left(1+\frac{\sqrt{\sum_{k=1}^{d}r_{j,k}^{2}}}{\sqrt{\lambda }}\right)}^{2}r_{j,i}^{2}$.

*Proof*. Since the inequality $\|w_{t}{\|}_{2}\leq \frac{1}{\sqrt{\lambda }}$ holds for any *t* ∈ [*T*], we have $sup_{w,u\in \chi }{\left\|w-u\right\|}_{2}\leq \frac{2}{\sqrt{\lambda }}$ and $sup_{w,u\in \chi }{\left\|w-u\right\|}_{\infty }\leq \frac{2}{\sqrt{\lambda }}$ according to the triangle inequality and ‖**w**‖_∞_ ≤ ‖**w**‖_2_.

Using Lemma1 and assumption ‖**x**‖_∞_ ≤ 1, we have *r*_*t*,*i*_ ≤ *max*_*j*<*t*_|**x**_*j*,*i*_ − **x**_*t*,*i*_| ≤ 2 and $sup_{w\in \chi }\|g_{t}{\left(w\right)\|}_{\infty }\leq \underset{i\leq d}{max}\left|g_{t,i}\right|\leq \sqrt{2\lambda +8{\left(1+2\sqrt{\frac{d}{\lambda }}\right)}^{2}}$. Then it is easy to obtain $sup_{w\in \chi }\|g_{t}{\left(w\right)\|}_{2}\leq \sqrt{d}\underset{i\leq d}{max}\left|g_{t,i}\right|\leq \sqrt{2d\lambda +8d{\left(1+2\sqrt{\frac{d}{\lambda }}\right)}^{2}}$.

According to the definitions of $\bar{v}$ and **g**_1:*T*,*i*_, we have

$$\begin{array}{ccc} \sum_{i=1}^{d}\sqrt{T{\bar{v}}_{T,i}} & = & \frac{\sum_{i=1}^{d}\sqrt{T\sum_{j=1}^{T}\left(1-\beta_{2}\right)\beta_{2}^{T-j}g_{j,i}^{2}}}{\sqrt{1-\beta_{2}^{T}}}\\ & \leq & \frac{\sum_{i=1}^{d}\sqrt{T\sum_{j=1}^{T}\left(1-\beta_{2}\right)\beta_{2}^{T-j}B_{j,i}}}{\sqrt{1-\beta_{2}^{T}}} \end{array}$$ (19)

$$\begin{array}{c} \sum_{i=1}^{d}{\left\|g_{1:T,i}\right\|}_{2}=\sum_{i=1}^{d}\sqrt{\sum_{t=1}^{T}g_{t,i}^{2}}\leq \sum_{i=1}^{d}\sqrt{\sum_{t=1}^{T}B_{j,i}} \end{array}$$ (20)

Plugging (19), (20), the bound of the gradient and the bound of the distance between any weight vectors into (17), we have

$$\begin{array}{c} \begin{array}{cc} R(T)\leq & \frac{2}{\lambda \eta (1-\beta_{1})}\frac{\sum_{i=1}^{d}\sqrt{T\sum_{j=1}^{T}\left(1-\beta_{2}\right)\beta_{2}^{T-j}B_{j,i}}}{\sqrt{1-\beta_{2}^{T}}}+\sum_{i=1}^{d}\frac{2\sqrt{2(1-\beta_{2})}\sqrt{\lambda +4{\left(1+2\sqrt{\frac{d}{\lambda }}\right)}^{2}}}{\eta \left(1-\beta_{1}\right){\left(1-\rho \right)}^{2}}\\ + & \frac{2\eta \left(1+\beta_{1}\right)\sqrt{\lambda +4{\left(1+2\sqrt{\frac{d}{\lambda }}\right)}^{2}}}{\left(1-\beta_{1}\right)\sqrt{1-\beta_{2}}{\left(1-\gamma \right)}^{2}}\sum_{i=1}^{d}\sqrt{\sum_{t=1}^{T}B_{j,i}} \end{array} \end{array}$$

which completes the proof.

If the features are rather dense, then we have

$$\begin{array}{ccc} B_{j,i} & = & 2{\left(\lambda w_{j,i}\right)}^{2}+2{\left(1+\frac{\sqrt{\sum_{k=1}^{d}r_{j,k}^{2}}}{\sqrt{\lambda }}\right)}^{2}r_{j,i}^{2}\\ & \leq & 2\lambda +8{\left(1+\frac{2\sqrt{d}}{\sqrt{\lambda }}\right)}^{2}. \end{array}$$ (21)

according to the inequality $\underset{i\leq d}{max}|w_{j,i}{\left|\leq \|w\right\|}_{2}\leq 1/\sqrt{\lambda }$ and *r*_*j*,*i*_ ≤ 2 for any *i* ∈ [*d*]

If we denote the constant $C=2\lambda +8{\left(1+\frac{2\sqrt{d}}{\sqrt{\lambda }}\right)}^{2}$, it is easy to obtain:

$$\begin{array}{ccc} \frac{\sum_{i=1}^{d}\sqrt{T\sum_{j=1}^{T}\left(1-\beta_{2}\right)\beta_{2}^{T-j}B_{j,i}}}{\sqrt{1-\beta_{2}^{T}}} & \leq & \frac{\sum_{i=1}^{d}\sqrt{T\sum_{j=1}^{T}\left(1-\beta_{2}\right)\beta_{2}^{T-j}C}}{\sqrt{1-\beta_{2}^{T}}}\\ & \leq & \frac{\sum_{i=1}^{d}\sqrt{T\sum_{j=1}^{T}\left(\beta_{2}^{T-j}-\beta_{2}^{T+1-j}\right)C}}{\sqrt{1-\beta_{2}^{T}}}\\ & = & \frac{\sum_{i=1}^{d}\sqrt{TC(\sum_{j=0}^{T-1}\beta_{2}^{j}-\sum_{j=1}^{T}\beta_{2}^{j})}}{\sqrt{1-\beta_{2}^{T}}}\\ & = & d\sqrt{TC} \end{array}$$ (22)

Also we have:

$$\begin{array}{c} \sum_{i=1}^{d}\sqrt{\sum_{t=1}^{T}B_{j,i}}\leq d\sqrt{TC}. \end{array}$$ (23)

Therefore, AdmOAM’s regret bound is $O(\sqrt{T})$ for the dense feature space and its convergence rate is $O(1/\sqrt{T})$ as in the general case of the non-adaptive algorithms.

When the features are sparse, the term *B*_*j*,*i*_ should be much smaller than *C*. Therefore, the regret bound of AdmOAM should be much smaller than $O(\sqrt{T})$, which results in a faster convergence. Besides, AdmOAM achieves comparable convergence rate with respect to AdaOAM according to [22].

From the above analysis, we can conclude that AdmOAM converges faster than the non-adaptive algorithms and stays in comparable convergence rate as AdmOAM.

## Experimental results

In this section, we evaluate the performance of AdmOAM on several standard benchmark datasets.

### Compared algorithms

Since we only concentrate on online scenarios, we do not take existing batch learning methods into consideration. We compare AdmOAM with 4 competing online AUC maximization algorithms:

- **OAM_**seq**_**: The OAM algorithm using sequential updating [19];
- **OAM_**gra**_**: The OAM algorithm using online gradient updating [19];
- **OPAUC**: One-pass AUC optimization algorithm [17];
- **AdaOAM**: The adaptive subgradient online AUC optimization algorithm [16].

### Experimental testbed and setup

We conduct the experiments on 13 benchmark datasets, which can be downloaded from the LIBSVM (http://www.csie.ntu.edu.tw/~cjlin/libsvmtools/) and the UCI websites (http://www.ics.uci.edu/~mlearn/MLRepository.html). Note that the glass, vehicle, dna and acoustic are multi-class datasets, we transform them into binary-class by randomly setting one class as the positive class, and the others as negative. The sparsity of the dataset is defined as the number of zero elements divided by the total number of elements in its feature matrix. Besides, the features have been rescaled to [−1, 1]. The details of the datasets are summarized in Table 1.

**Table 1:** Information about datasets.

Datasets	instances	features	*T*_−_/*T*_+_	sparsity(%)
glass	214	9	2.0571	0.1558
svmguide4	300	10	5.8182	0.0333
heart	690	14	1.2500	3.7607
australian	690	14	1.2476	12.5569
vehicle	846	18	2.9906	1.9766
german	1,000	24	2.3333	4.1625
svmguide3	1,243	22	3.1993	0.3730
w1a	2,477	300	33.4027	96.1768
dna	3,186	180	1.0796	74.7329
a9a	32,561	123	3.1526	88.7243
cod-rna	59,535	8	2.0000	0.0015
acoustic	78,823	50	3.3165	0
ijcnn1	141,691	22	9.4453	0

We conduct nested cross-validation for hyperparameter searching and model evaluation. In the outer cross-validation, we conduct 5-fold cross-validation on each benchmark dataset, where 4 folds are for training and the remaining fold is treated as the test set. In the inner cross-validation, we apply 5-fold cross-validation on the training set for hyperparameter searching. After the process of the inner cross-validation, we train the model on the whole training set with the tuned hyperparameters. Finally, we calculate the AUC score of the model on the test set for model evaluation. For further reducing the variance in the results, we apply 4 independent 5-fold nested cross-validation on each dataset. Therefore the AUC performance of each algorithm on different datasets is the average over 20 independent runs. For the hyperparameter searching, we tune the learning rate *η* ∈ 2^[−10:1:3]^ and the regularization parameter λ ∈ 2^[−10:1:3]^ for AdmOAM, AdaOAM and OPAUC. For the exponential decay rates of AdmOAM, we decide *β*_1_ ∈ [0.1: 0.1: 0.9] and *β*_2_ ∈ [0.099: 0.1: 0.999]. For buffer sampling algorithms like OAM_*seq*_ and OAM_*gra*_, we tune the penalty parameter *C* ∈ 2^[−10:1:10]^, and the size of the buffer is set at 100 as recommended in [19].

### Evaluation on benchmark datasets

In this subsection, we analyse the average AUC values, convergence rate and running time of AdmOAM with compared methods. Table 2 shows the average AUC values over 20 independent runs on 13 benchmark datasets.

**Table 2:** Evaluation on benchmark datasets for comparing AUC performance (mean+std).

datasets	AdmOAM	AdaOAM	OPAUC	OAM_*seq*_	OAM_*gra*_
glass	.8253±.0554	.8144±.0500	.7966±.0722	.8062±.0731	.7407±.0755
svmguide4	.9491±.0245	.8429±.0434	.8006±.0657	.9040±.0386	.9091±.0569
heart	.9111±.0299	.9083±.0315	.9073±.0586	.8024±.0767	.8678±.0586
australian	.9304±.0218	.9285±.0206	.9269±.0210	.8683±.0278	.8252±.0643
vehicle	.8316±.0240	.7927±.0184	.7854±.0285	.7170±.0572	.7469±.0634
german	.8003±.0216	.7990±.0227	.7963±.0249	.7544±.0300	.7675±.0320
svmguide3	.7527±.0382	.7434±.0343	.7315±.0399	.7003±.0541	.6900±.0460
w1a	.9439±.0222	.9317±.0355	.9184±.0391	.8891±.0570	.8978±.0528
dna	.9840±.0033	.9826±.0031	.9832±.0032	.9622±.0050	.9620±.0052
a9a	.9003±.0039	.9002±.0039	.8998±.0039	.8459±.0138	.8469±.0150
cod-rna	.9762±.0011	.9740±.0012	.9701±.0012	.8578±.0662	.8256±.0607
acoustic	.8933±.0016	.8907±.0019	.8901±.0018	.8389±.0168	.8408±.0165
ijcnn1	.9094±.0027	.8976±.0028	.8471±.0025	.8441±.0419	.8766±.0396

Based on the results in Table 2, we have several observations. Firstly, the two adaptive methods AdmOAM and AdaOAM achieve higher AUC score than the other three non-adaptive methods in most cases. Therefore, the adaptive learning strategy can effectively improve the performance of the existing online AUC maximization algorithms. Secondly, AdmOAM obtains better or comparable performance than AdaOAM for most datasets. Especially in the svmguide4 and vehicle datasets, AdmOAM achieves much higher AUC scores than AdaOAM. This indicates the effectiveness of AdmOAM over AdaOAM.

**Fig 1:**
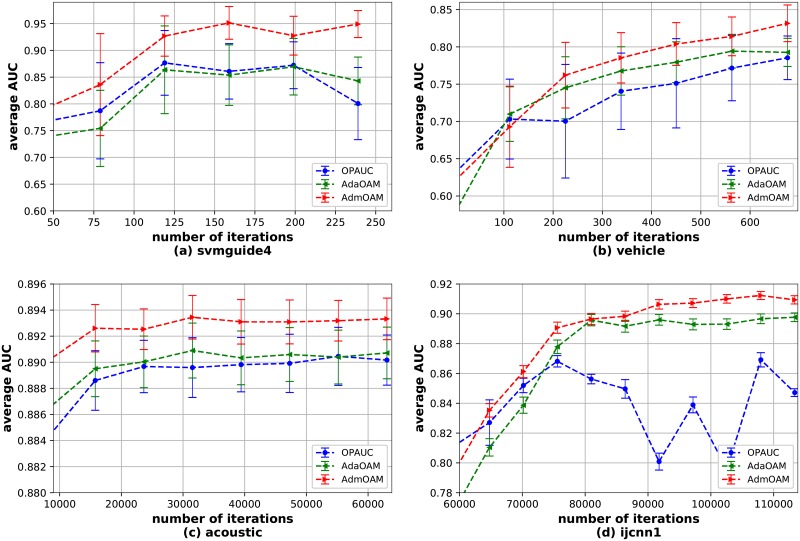
Evaluation of convergence rate on benchmark datasets.

Next we provide the analysis on the speed of the convergence of AdmOAM. For each online AUC maximization algorithm, it updates the model from a sequence of training data one at a time. For comparing the convergence speed, we evaluate the AUC score of different online learning algorithms on the testing set. Compared with reservoir sampling methods like OAM_*seq*_ and OAM_*gra*_, the algorithms based on the one-pass learning mode obtain better performance according to the results in Table 2. Therefore, we compare the convergence rate of AdmOAM with AdaOAM and OPAUC. Fig 1(a)–1(d) depict the convergence curves on 4 benchmark datasets with the error bars. Specifically, we report the average AUC score across 20 independent runs on the testing datasets at different iterations. From Fig 1, we can observe that AdmOAM converges faster than the other two algorithms. With the increasing of the number of iterations, AdmOAM achieves a higher AUC score than AdaOAM and OPAUC. This validates our theoretical analysis and demonstrates the effectiveness of AdmOAM.

**Fig 2:**
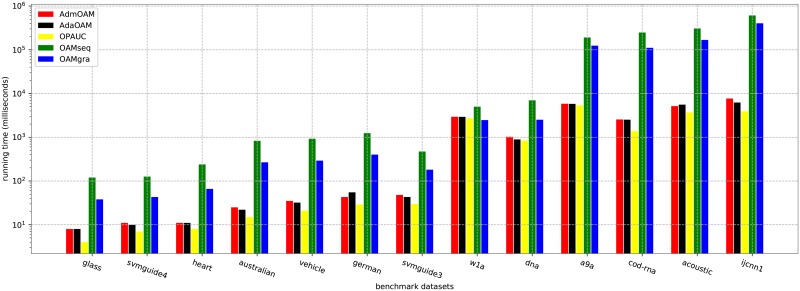
Comparsion of the runing time (in milliseconds). The *y*-axis is set as log-scale.

We also present the running time on 13 datasets in Fig 2. On most datasets, AdmOAM is more efficient than OAM_*seq*_ and OAM_*gra*_, and stays competitive with AdaOAM in the computational complexity. Compared to OPAUC, AdmOAM needs to spend a little more time for updating two extra vectors of the first and second moments.

### Evaluation of parameter sensitivity

**Fig 3:**
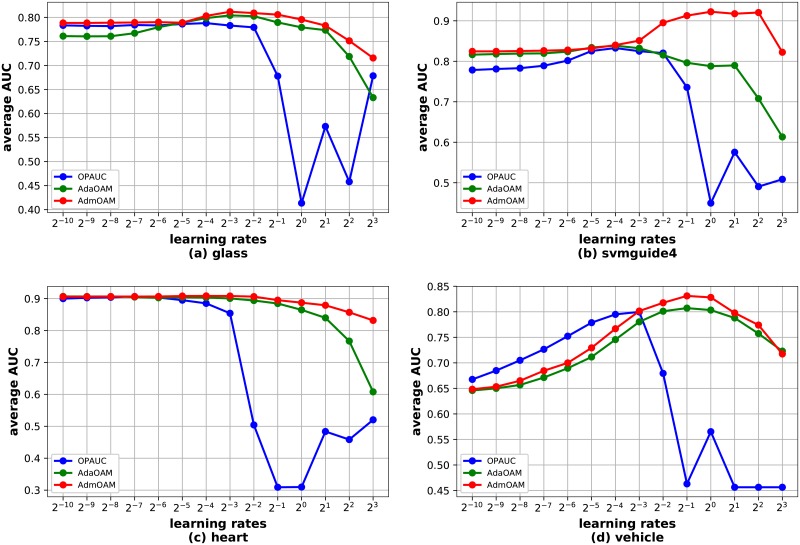
Evaluation of parameter sensitivity.

Since AdmOAM adaptively updates the learning rates, we mainly focus on the parameter sensitivity of learning rate and the other parameters are fixed at the tuned values. We report the average test AUC score across 5 independent runs (1 trail of 5-fold cross-validation) with the range of learning rates *η* ∈ 2^[−10:1:3]^ in Fig 3. Based on the results in Fig 3, we can observe that AdmOAM is less sensitive to the learning rate than OPAUC especially when the value of the learning rate is over 2^−2^. In [16], the author claimed that AdaOAM is insensitive to the parameter settings. From Fig 3, we can observe that AdmOAM obtains comparable or better average AUC score than AdaOAM. The above results indicate that AdmOAM can effectively adjust its per-coordinate learning rate and is less sensitive to the parameter settings.

## Conclusion and future work

In this paper we proposed AdmOAM, an Adaptive Moment estimation method for Online AUC Maximization. It applies the estimation of moments of gradients to accelerate the convergence and mitigate the rapid decay of the learning rates. Theoretically, we have analysed the regret bound of the proposed algorithm. It can achieve a lower bound than non-adaptive online AUC maximization algorithms and stay competitive to AdaOAM. Moreover, we evaluated its performance with several competing algorithms on benchmark datasets. The experimental results validate the theoretical analysis and indicate the effectiveness of the proposed algorithm.

For future work, there are several research directions. Firstly, AdmOAM uses all features for AUC maximization. This is not efficient and scalable for high-dimensional sparse datasets. It would be interesting to combine AdmOAM with feature selection techniques for learning a sparse model. Secondly, AdmOAM is not suitable for the non-linearly separable data with linear model. It would be interesting to combine AdmOAM with online kernel learning methods for handling the nonlinearity of the data.
